# Characterization of Cell Wall Proteins in *Saccharomyces cerevisiae* Clinical Isolates Elucidates Hsp150p in Virulence

**DOI:** 10.1371/journal.pone.0135174

**Published:** 2015-08-13

**Authors:** Pang-Hung Hsu, Pei-Chi Chiang, Chia-Hsun Liu, Ya-Wen Chang

**Affiliations:** 1 Department of Bioscience and Biotechnology, National Taiwan Ocean University, Keelung, Taiwan; 2 Department of Clinical Laboratory Sciences and Medical Biotechnology, National Taiwan University, Taipei, Taiwan; 3 Department of Laboratory Medicine, National Taiwan University Hospital, Taipei, Taiwan; National University of Ireland Maynooth, IRELAND

## Abstract

The budding yeast *Saccharomyces cerevisiae* has recently been described as an emerging opportunistic fungal pathogen. Fungal cell wall mannoproteins have been demonstrated to be involved in adhesion to inert surfaces and might be engaged in virulence. In this study, we observed four clinical isolates of *S*. *cerevisiae* with relatively hydrophobic cell surfaces. Yeast cell wall subproteome was evaluated quantitatively by liquid chromatography/tandem mass spectrometry. We identified totally 25 cell wall proteins (CWPs) from log-phase cells, within which 15 CWPs were quantified. The abundance of Scw10p, Pst1p, and Hsp150p/Pir2p were at least 2 folds higher in the clinical isolates than in S288c lab strain. Hsp150p is one of the members in Pir family conserved in pathogenic fungi *Candida glabrata* and *Candida albicans*. Overexpression of Hsp150p in lab strain increased cell wall integrity and potentially enhanced the virulence of yeast. Altogether, these results demonstrated that quantitative cell wall subproteome was analyzed in clinical isolates of *S*. *cerevisiae*, and several CWPs, especially Hsp150p, were found to be expressed at higher levels which presumably contribute to strain virulence and fungal pathogenicity.

## Introduction

Yeast *Saccharomyces cerevisiae* was considered to be a harmless microorganism; however, occasional reports of uncommon infections that were attributed to *S*. *cerevisiae* were revealed since 1980 [1–6]. The clinical isolates of *S*. *cerevisiae* were first genetically characterized as pathogens in 1994 [7]. Some clinical isolates showed pathogenic potential by proliferating and causing death in DBA/2 mice, but non-clinical yeast strains did not [8, 9]. Therefore, *S*. *cerevisiae* has been regarded as one of emerging opportunistic yeast infections [10–14]. Several virulence factors have been addressed between clinical and laboratory strains of *S*. *cerevisiae*, such as thermotolerance and pseudohyphal formation [7]; however, the correlation between these traits and the composition of the cell wall proteome requires further elucidation.

Yeast cell walls are essential for the survival of fungal cells. Two primary components of the inner part of *S*. *cerevisiae* cell wall, β-glucan and chitin, are served as scaffold and recognized by specialized innate immune receptors [15, 16]. It has been demonstrated that the predominant yeast cell wall molecule, β-glucan, is a potent immunostimulatory molecule [17]. In addition, mannoproteins, mainly glycosylphosphatidylinositol (GPI)-modified CWPs and alkali-sensitive linkage (ASL)-CWPs located at the outer part of cell wall, have been demonstrated to be involved in adhesion to host cells, virulence, fungal morphogenesis, cell wall biogenesis, and biofilm formation [18–23]. The association between cell surface polysaccharides and immune recognition of fungi also suggested that the changes in yeast cell surface properties may be responsible for increased virulence [15, 16, 24, 25]. Mutants with changes in the composition of cell walls may be more virulent because of the altered cell surface that leads to misrecognition by the innate immune system and stimulation of proinflammatory cytokines such as TNF-α, IL-1β, and IL-6 [26]. It has been shown that overproduction of proinflammatory cytokines, such as TNF-α stimulated by hypervirulent yeasts, is associated with septic shock in mice [26, 27].In addition, a similar overall cell wall structure exists in related fungi, such as human pathogens *Candida albicans* and *Candida glabrata* [28, 29]. Therefore, deciphering the cell surface architecture of clinical isolates of *S*. *cerevisiae* may highlight new determinants of fungal virulence.

Mass spectrometry-based proteomics has provided a powerful tool for systematic elucidation of yeast *S*. *cerevisiae* proteome. SILAC (stable isotope labeling by amino acids in cell culture) labeling of yeast was applied to generate one-to-one pairs of peptide signals which facilitates a highly reliable measurement of relative and absolute protein amounts [30–32]. Here we used the auxotrophic lab strain for stable isotope incorporation, and further compared its cell wall protein profile quantitatively to the clinical isolates. We found that Scw10p, Pst1p, and Pir family proteins, such as Pir1p, Hsp150p/Pir2p, and Cis3/Pir4p, were expressed significantly higher in clinical isolates than in S288c lab strain. In particular, high levels of Hsp150p enhanced cell wall integrity and the ability of adherence to polystyrene surface. Strains overexpressed *HSP150* were more potent to elicit proinflammatory cytokine TNF-α from macrophages, suggesting that alteration of cell wall composition and architecture causes yeast hypervirulent.

## Materials and Methods

### Strains and Media

Strains were grown in YPD (1% (w/v) yeast extract, 2% (w/v) bactopeptone, and 2% (w/v) glucose) media and harvested at A_600_ = 0.8–1.2. YYC1-3 were isolated and obtained from National Taiwan University Hospital, Taipei, Taiwan. YJM309 (YYC38) was the gift from John McCusker of Duke University Medical Center, North Carolina [7]. Σ1278b (YYC304) was obtained from Fang-Jen Lee at National Taiwan University, Taipei, Taiwan. YYC377 (MAT a/α *his3Δ1/ his3Δ1 leu2Δ0/ leu2Δ0 LYS2/lys2Δ0 MET15/met15Δ0 ura3Δ0/ ura3Δ0*) is the diploid strain in S288c background generated by mating BY4741 and BY4742, the parental strains of Yeast Deletion Project obtained from Wen-Hsiung Li at Academia Sinica, Taipei, Taiwan. YYC370 (MAT a/α *ADE2/ade2Δ*::*hisG HIS3/his3Δ200 leu2Δ0/leu2Δ0 lys2Δ0/lys2Δ0 MET15/met15Δ0 TRP1/trp1Δ63 URA3/ ura3Δ0*) is also the diploid strain in S288c background generated by mating BY4704 and BY4739 obtained from Wen-Hsiung Li at Academia Sinica, Taipei, Taiwan. The *hsp150* deletion strain in BY4741 background (MAT a *his3Δ1 leu2Δ0 met15Δ0 ura3Δ0 hsp150Δ*::*KanMX4*) was obtained from Wen-Hsiung Li at Academia Sinica, Taipei, Taiwan. For Calcofluor white (CFW) or Congo Red (CR) sensitivity assay, overnight cultures were diluted and spotted to YPD plates with 50 μg/mL CFW or 100μg/mL CR and incubated at 30°C for 3 days.

YYC173 is the segregant of YYC1. *HSP150-173* and *HSP150-288* alleles were amplified by PCR from YYC173 and S288c strains respectively with a Myc tag inserted into the end of the ORFs. The PCR products were digested with restriction enzymes *Bam*HI and *Sal*I and then subcloned into the vector pRS415-*GAL1*, which is the gift from Tien-Hsien Chang at Academia Sinica, Taipei, Taiwan. The constructs were transformed into *hsp150Δ* deletion strain, and the expression of *HSP150* alleles were driven by *GAL1* promoter by growing in SC-galactose media (0.67% (w/v) yeast nitrogen base and 2% (w/v) galactose). For CFW or CR sensitivity assay, overnight cultures were diluted and spotted to SC-galactose plates with 500 μg/mL CFW or 150 μg/mL CR and incubated at 30°C for 3 days.

### Cell Hydrophobicity Assay

Determination of cell surface hydrophobicity using MATH (microbial adhesion to hydrocarbons) test was performed as described before [33], with some modifications. Yeast cells were grown in YPD media for overnight, washed in distilled water, and resuspended in 10 mM potassium phosphate buffer (pH 4) so that the basic OD_600_ of the suspension (A_0_) was 1.0. 80 μL *o*-xylene was added to a 800 μL suspension, vortexed at top speed for 1 min, and allowed to settle for 10 min. The OD_600_ of the aqueous phase (A_t_) was measured. The hydrophobicity was calculated by the percentage of cells adhered to *o*-xylene: Adhesion to xylene (%) = (1- A_t_ /A_0_) x 100. The strains were considered more hydrophobic if the percentage of adhesion is higher. Statistical significance was measured by Student’s *t* test.

### Adhesion Assay

Adherence of *S*. *cerevisiae* to polystyrene surface was performed as describe before [34, 35]. Strains were harvested and washed with distilled water twice, and then resuspended to 1.0 OD_600_ in YPD media with 0.1% glucose. 100 μL of cell suspension was transferred into wells of a 96-well polystyrene plate (SPL Life Sciences, Korea) and incubated at 30°C for 1 hr. Cell suspension was then removed and cells adhere to polystyrene were stained with 100 μL 1% (w/v) crystal violet for 15 min [35]. The wells were washed repeatedly with distilled water, and 100 μL of 33.3% acetic acid were added to release bound crystal violet. The absorbance was measure at 590 nm. Statistical significance was measured by Student’s *t* test.

### SILAC Labeling

SILAC labeling was performed as described before [30] with modifications. Clinical isolates YYC1-3, YYC38, and YYC 370 were grown in “light” SD-glucose media (0.67% (w/v) yeast nitrogen base, 2% (w/v) glucose, and amino acid supplements 50 mg/L adenine, 20 mg/L L-histidine, 100 mg/L L-leucine, 20 mg/L methionine, 20 mg/L L-tryptophan, and 20 mg/L uracil) with additional 30 mg/L normal L-lysine (Sigma). YYC370 were grown in “heavy” SD-glucose media, which is the SD-glucose media but with 30 mg/L L-lysine (4,4,5,5-D4) (Cambridge Isotope Laboratories, Inc.) instead of normal L-lysine. Either light or heavy SILAC-labeled yeast cells were grown for more than 20 generations and harvested at A_600_ = 0.8–1.2. Equal amounts of each light and heavy cells (as determined by A_600_ measurement) were then mixed 1:1, harvested and washed for cell wall preparation. The mixture of equal amounts of light and heavy YYC370 was served as controls of our quantification later.

### Cell Wall Isolation

Cell walls were isolated as described [36, 37]. Briefly, mid-log phase of cells were harvested and resuspended in 10 mM Tris-HCl, pH 7.5. Cells were fully broken with 0.55-mm glass beads (Thomas Scientific, USA) in the presence of protease inhibitor (Roche). Cell wall debris was obtained by centrifugation at 4,500xg for 15 min. To remove noncovalently linked proteins and intracellular contaminants, isolated cell walls were washed extensively with 1 M NaCl and then resuspended in extraction buffer (2% SDS, 100 mM Na-EDTA, 40mM β-mercaptoethanol, and 50 mM Tris-HCl, pH 7.8) and boiled for 5 min at 100°C. The extraction procedures were repeated, and SDS-extracted walls were washed three times with water and stored at -20°C until use.

### LC-MS/MS Analysis and SILAC Quantitation

Peptides for LC-MS/MS analysis were obtained from the proteolytic digestion directly from isolated cell wall fractions. 5 biological replicates of proteomics analyses were performed. The SDS-extracted cell walls were reduced by dithiothreitol (10 mM) at 55°C for 30 min followed by alkylation by iodoacetamide (50 mM) at room temperature for 30 min in the dark. Cell wall fractions were digested overnight by sequencing grade trypsin. After enzymatic reaction, peptides in the supernatants were collected and dried under vacuum.

Nano-LC-MS/MS experiments were performed on a LTQ-FT mass spectrometer (Thermo Scientific, Inc., Waltham, MA) with a nano-electrospray ion source (New Objective, Inc., Woburn, MA) in positive ion mode. The liquid chromatographic separation was using Agilent 1100 Series binary high-performance liquid chromatography system (Agilent Technologies, Palo Alto, CA) with Famos autosampler (LC Packings, San Francisco, CA). Peptide samples were injected onto a self-packed pre-column (150 μm I.D. x 20 mm, 5μm, 200 Å) followed by a self-packed reversed phase C18 nano-column (75 μm I.D. x 300 mm, 5μm, 100 Å) for chromatographic separation. A linear gradient of 5 to 40% mobile phase B (0.1% formic acid in 80% acetonitrile) was applied for 100 min at a flow rate of 300 nL/min. The positive ion mode was used in MS analysis with electrospray voltage of 2 kV and capillary temperature at 200°C. A MS scan cycle was initiated with a survey MS spectrum (m/z 300–2000) performed on the FT-ICR mass spectrometer with resolution of 100,000 at 400 Da. Ten most abundant ions detected in this scan were subjected to a MS/MS experiment performed in the LTQ mass spectrometer. Ion accumulation (Auto Gain Control target number) and maximal ion accumulation time for survey MS and MS/MS were set at 1 x 10^6^ ions, 1000 ms and 5 x 10^4^ ions, 200 ms respectively. Ions were fragmented by use of CID (collision induced dissociation) with the following parameters: the normalized collision energy to 35%, the activation Q to 0.3, and the activation time to 30 ms.

For data analysis, all MS/MS spectra were converted to mzXML format from experiment RAW files by MM File Conversion Tools (http://www.massmatrix.net [38]), and then analyzed by MassMatrix [39] for MS/MS ion search as well as SILAC quantitation. Searches were performed with a precursor mass tolerance of 10 ppm and a fragment mass tolerance of 0.6 Da. Trypsin was assigned as the enzyme for data analysis with the miss cleavage number three. The oxidation of methionine, carbamidomethylation of cysteine, phosphorylation of serine/threonine/tyrosine, and acetylation of lysine were assigned as variable modifications in search parameters. Relative quantification results were directly outputted from MassMatrix and compared to two other methods, the automatic quantification by MaxQuant and manual quantification. The statistical significance was determined by two-way ANOVA.

### Macrophage Stimulation and TNF-α Measurement

Murine RAW 264.7 macrophage cell line was a gift from Betty Wu-Hsieh at National Taiwan University, Taipei, Taiwan, originally obtained from the American Type Culture Collection, Rockville, Md., and cultured in DMEM-10 (DMEM (Biological Industries, Israel) with 10% heat-inactivated FCS (GE Healthcare), 1x of Hyclone non-essential amino acids (GE Healthcare), 100 unit/mL penicillin, 0.1 mg/mL streptomycin, and 250 ng/mL amphotericin B) [40, 41]. Yeast cells were added at a yeast to macrophage ratio of 60 and allowed to bind and phagocytosed for 30 min at 37°C. Macrophages were washed twice with PBS to remove unbound yeast and incubated in 0.5 mL of DMEM-10 for 6 hr. Murine TNF-α levels in culture supernatants were measured by ELISA according to the manufacturer’s instructions (R & D system). Infections were done in duplicate or triplicate and ELISA measurements were taken in duplicate or triplicate. Statistical significance was measured using Student’s *t* test.

## Results and Discussion

### Clinical Isolates of *S*. *cerevisiae* Were More Hydrophobic and Capable of Adhering to Plastic Surfaces

**Fig 1:**
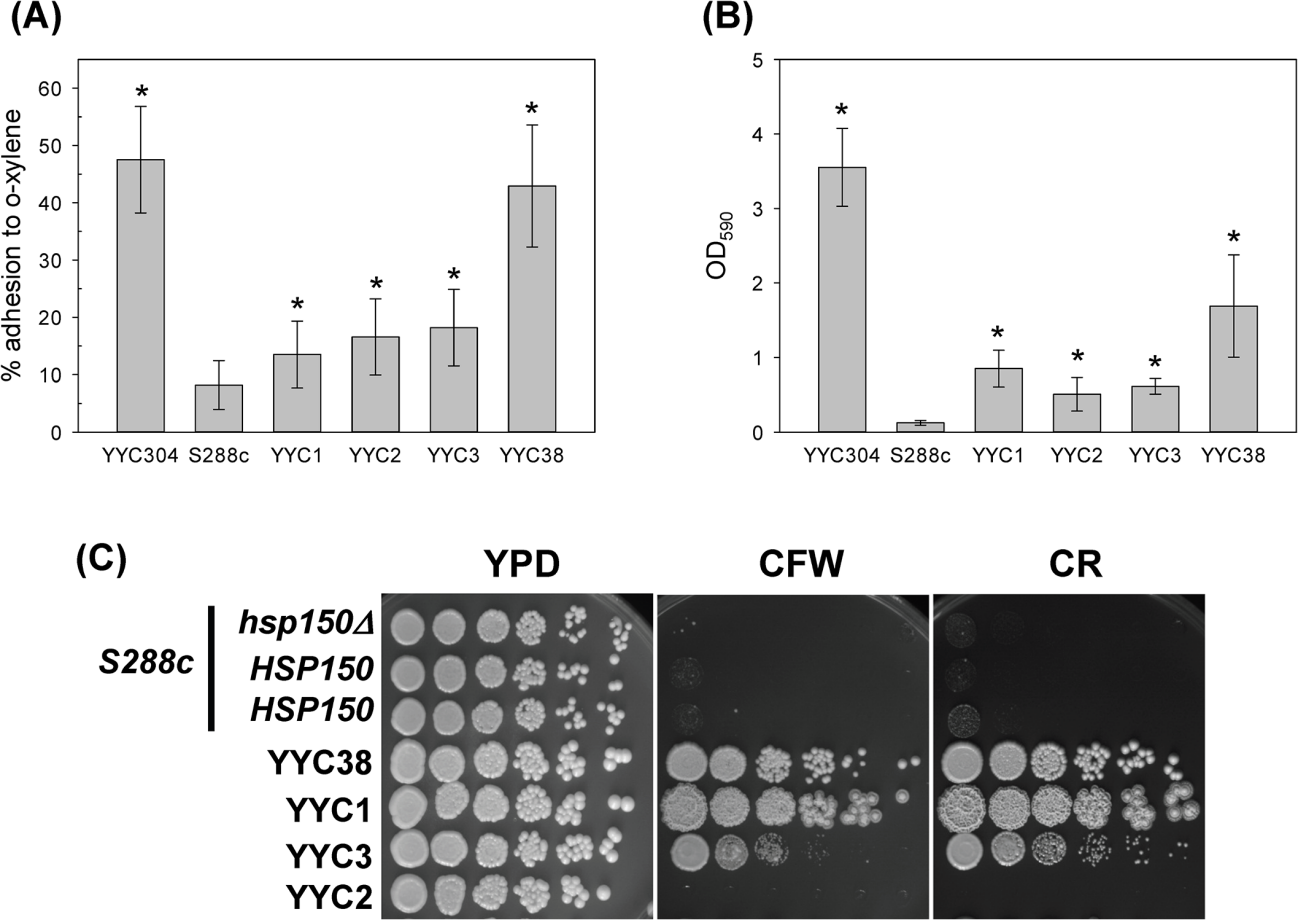
The *S*. *cerevisiae* clinical isolates showed hydrophobic cellular surface and resistant to cell wall-perturbing agents. Strains S288c and YYC304 were served as negative and positive controls respectively. The clinical isolates YYCs 1–3 and 38 were subjected to cell hydrophobicity assay (A), adhesion assay (B), and sensitivity assay with 50 μg/mL CFW or 100 μg/mL CR (C) as described. Serial 5-fold dilutions of each strain were spotted on solid YPD media or in the presence of CFW or CR. The plates were incubated at 30°C for 3 days. 3 biological replicates were performed. Asterisks indicate statistically significant differences from S288c (*p* < 0.05).

Yeast cellular surface hydrophobicity has been shown as a potentially important pathogenic factor involved in adherence and biofilm formation [42]. First we investigated the cell surface characteristics between the laboratory strain and the clinical isolates of *S*. *cerevisiae* by the MATH test, in which the partition of cells in *o*-xylene represents a quantitative measurement of hydrophobicity [33, 43]. While hydrophobic strain YYC304 in Σ1278b background and clinical isolate YYC38 showed that about 43–45% of cells adhered to *o*-xylene, the clinical isolates YYC1-3 all showed 13–18% of adherence, about increased up to 1.5 to 2 folds comparing to lab strain S288c (Fig 1A). It indicated that all four clinical strains tested showed significantly hydrophobic cell surfaces, while the S288c laboratory strain was relatively hydrophilic.

On the other hand, cell surface property also affects the adherence to polystyrene plates. Attached cells were stained with crystal violet and absorbance at 590 nm were measured proportional to cell numbers. We found that the mount of cells adhesive to polystyrene surface was dramatically increased up to 4.2–14 folds in all four clinical strains tested comparing to the S288c laboratory strain (Fig 1B). To further investigate the difference of cell wall structure and integrity between the lab strains and clinical isolates, we examined strain sensitivity to cell wall-perturbing agents CR and CFW, which interfere with construction and biosynthesis of the main architecture of cell wall. Wild-type S288c strain showed hypersensitive to high concentration of 100 μg/ml of CR or 50 μg/ml of CFW, while three of the clinical isolates except YYC2 were much more resistant to these two agents (Fig 1C). All these results indicated that the cell wall characteristics of the clinical isolates of *S*. *cerevisiae* recognizably vary from S288c.

### Comprehensive Mass-Spectrometry-Based Proteome Quantification of CWPs

To investigate the variation of fungal cell wall proteomics between the lab strain and the clinical isolates, we established analyses of *S*. *cerevisiae* CWPs by mass spectrometry. Cells growing into mid-log phase were collected, and SDS-treated cell walls were subjected to trypsin digestion to obtain peptide fragments which were further separated and sequenced by LC-MS/MS. Totally 25 CWPs were successfully identified from log-phase cells, including 15 GPI-modified proteins, 7 ASL-CWPs, and 3 other CWPs: Bgl2p, Ssa1p, and Tdh3p (Table 1), which are comparative to previous results [37]. However, since CWPs are potentially highly glycosylated, we cannot rule out that proteins would be missed if the trypsinized fragments fall out of the range detectable by LC-MS/MS in our analysis. Orthologs in *C*. *albicans* or *C*. *glabrata* for each protein were found based on *Candida* Genome Database (http://www.candidagenome.org/) except Cwp1p, Mkc7p, and Pry3p. Bgl2p is characterized as a beta-1,3-glucanase which introduces intrachain linkages and is involved in cell wall structure maintenance [44, 45]. Ssa1p is a chaperone protein belonging to HSP70 family. Although most of the Ssa proteins were found in the cytosol, it has been reported that Ssa1p was also detected in the cell wall [46]. Tdh3p is one of the isozymes of glyceraldehyde-3-phosphate dehydrogenases (GAPDHs). It has been interestingly detected in both the cytoplasm and cell wall [47].

**Table 1:** There were totally 25 CWPs identified by LC-MS/MS analysis.

Standard Name	Function	Homolog in *Candida* species	# of peptides Quantified/Detected
**GPI-CWP**			
Ccw12p	Cell wall mannoprotein	Pga62p (Ca)	0 / 1
Ccw14p/Ssr1p	Cell wall glycoprotein	Ssr1p (Ca, Cg)	4 / 4
Crh1p	Chitin transglycosylase	Crh11p (Ca); Crh1p (Cg)	9 / 12
Crh2p/Utr2p	Chitin transglycosylase	Utr2p (Cg)	2 / 3
Cwp1p	Cell wall mannoprotein	-	7 / 7
Cwp2p	Cell wall mannoprotein	Tir1p; Tir2p (Cg)	0 / 1
Ecm33p	Unknown	Ecm33p; Pst1 (Cg)	5 / 5
Exg2p	Exo-1,3-beta-glucanase	CAGL0M08756g (Cg)	0 / 1
Gas1p	β-1,3-glucanosyltransferase	Gas1p, Gas2p (Cg); Phr1p, Phr2p (Ca)	7 / 11
Gas3p	β-1,3-glucanosyltransferase	Gas4p (Cg)	4 / 9
Gas5p	β-1,3-glucanosyltransferase	Gas5p (Cg); Pga4p (Ca)	3 / 6
Mkc7p	Aspartyl protease	-	0 / 1
Plb2p	Phospholipase B	Plb2p (Cg); Plb3p (Ca)	0 / 1
Pry3p	Cell wall-associated protein	-	0 / 1
Pst1p	Cell wall protein	Pst1p; Ecm33p (Cg)	1 / 3
**ASL-CWP**			
Pir1p	O-glycosylated protein	Pir1p; Pir2p; Pir3p (Cg)	2 / 3
Pir2p/Hsp150p	O-mannosylated heat shock protein	Pir1p; Pir2p; Pir3p; Pir4p; Pir5p (Cg)	2 / 3
Pir3p	O-glycosylated protein	Pir1p; Pir2p; Pir3p; Pir4p (Cg)	0 / 1
Pir4p/Cis3p	Mannose-containing glycoprotein	Pir1p; Pir2p; Pir3p; Pir4p (Cg)	6 / 7
Scw4p	Glucanase	Scw4p; MP65 (Cg); MP65 (Ca)	3 / 10
Scw10p	Glucanase	Scw4p; MP65 (Cg); MP65 (Ca)	4 / 6
Tos1p	Cell wall protein	CAGL0M05599g (Cg); Tos1p (Ca)	3 / 3
**Others**			
Bgl2p	Endo-beta-1,3-glucanase	CAGL0G00220g (Cg); Bgl2p (Ca)	0 / 1
Tdh3p	Glyceraldehyde-3-phosphate dehydrogenase	CAGL0J00451g, Tdh3p (Cg); Tdh3p (Ca);	0 / 12
Ssa1p	ATPase	Ssa1p; Ssa3p (Cg); Ssa2p; Hsp70p (Ca)	0 / 11

The possible function and the homologs in *C*. *albicans* or *C*. *glabrata* for each characterized protein were listed. The peptide numbers used for quantification vs. protein identification were listed in the last column. EF-1α, Pdc1p, and Pma1p were internal controls to normalize the quantification results. Ca, *Candida albicans*; Cg, *Candida glabrata*.

**Fig 2:**
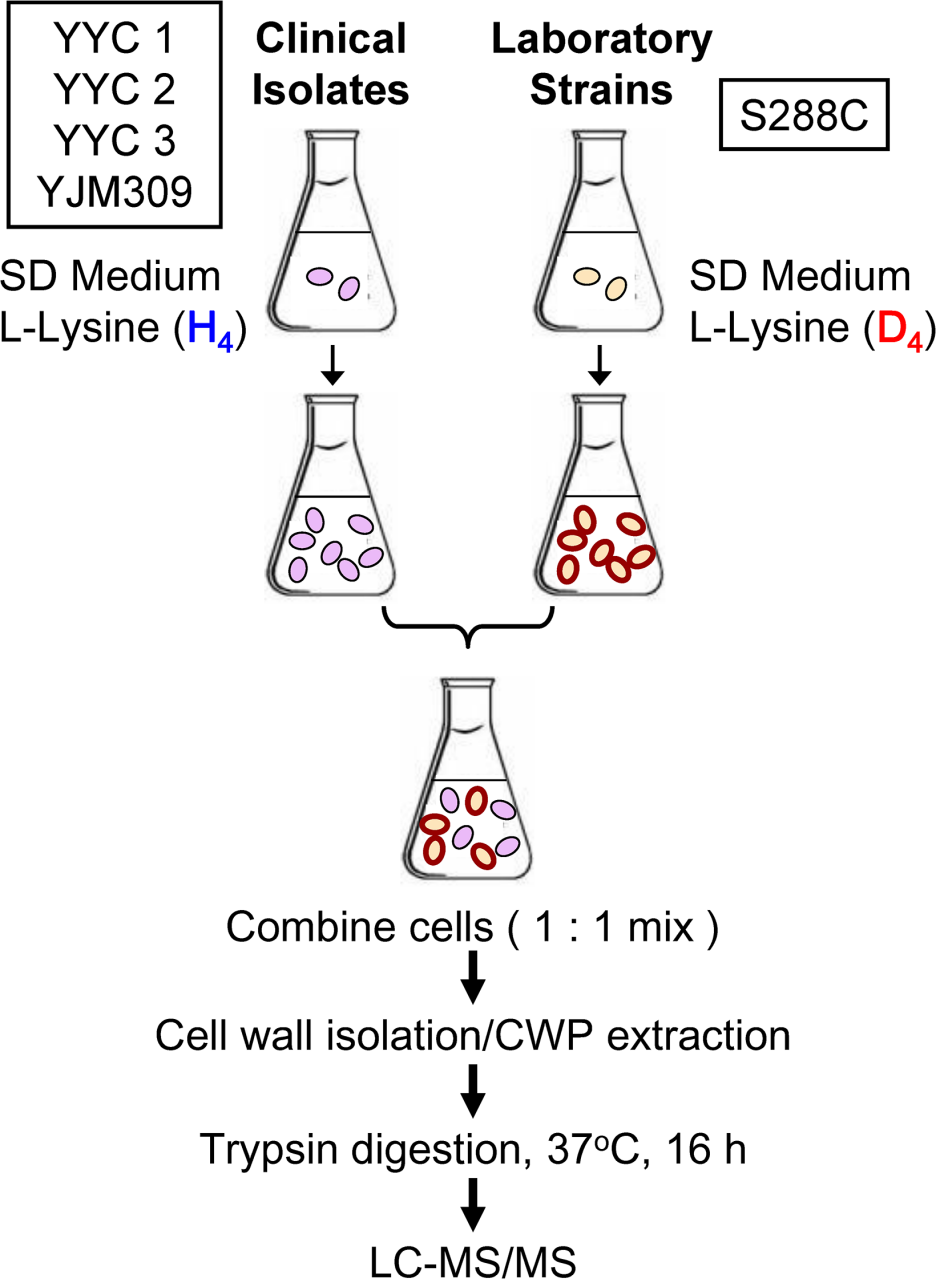
Strategy used to identify and quantify *S*. *cerevisiae* cell wall proteome. SILAC labeling followed by Nano-LC-MS/MS was used for relative quantitation.

**Fig 3:**
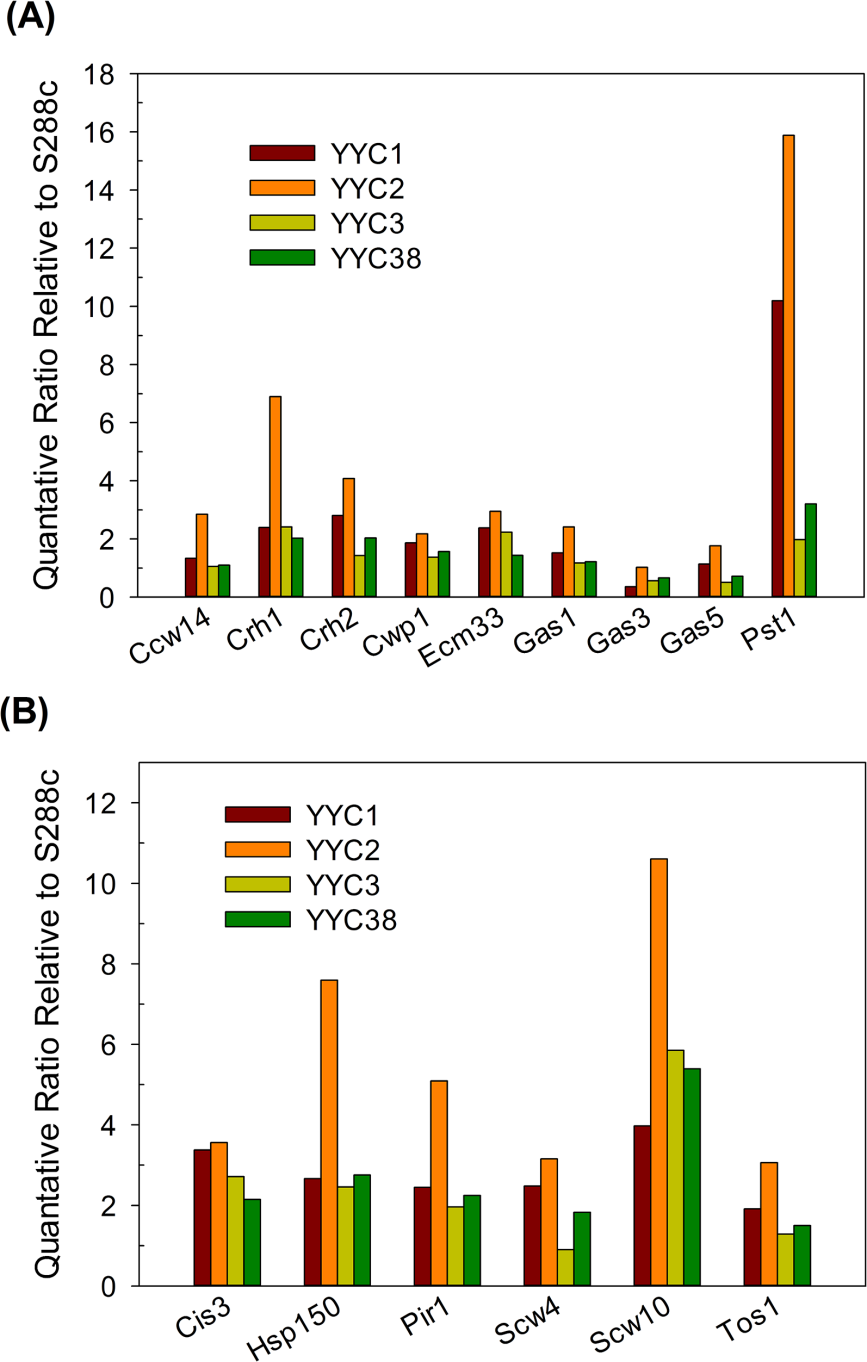
Scw10p, Pst1p and Hsp150p were expressed at significantly higher levels in the clinical isolates than in S288c. The protein levels in clinical isolates (YYC1-3 and 38) relative to S288c strain YYC370 were presented after normalization to the internal controls EF1-α, Pdc1p, and Pma1p. (A) GPI-CWPs and (B) ASL-CWPs. 5 biological replicates were performed and the averages were shown here.

To further quantitate the covalently linked CWPs between the clinical and lab strains, SILAC labeling followed by LC-MS/MS was used as an easy and accurate approach for CWP relative quantitation. The experimental scheme was illustrated in Fig 2, and the details were described in Materials and Methods. The S288c lab strain YYC370 was labeled with stable isotope incorporated amino acid L-lysine (4,4,5,5-D4) supplemented in growth media. In contrast, each of the clinical isolates YYC1, 2, 3, and 38 was cultured in the growth media containing regular amino acids. Equal cell numbers of S288c (heavy) and a clinical strain (light) were mixed together for CWPs isolation followed by trypsin digestion. Tryptic peptides were subjected to LC-MS/MS analyses, and peptide sequences and their abundances were further determined by MassMatrix. 15 of 25 CWPs were qualified for quantitation and the ratios of each CWP levels in particular clinical strain were calculated relative to those in S288c as shown in Fig 3. Similar results were obtained by MaxQuant automatic quantification or manual quantification (Supporting Information). The numbers and sequences of peptides detected and subjected to further quantification were listed in Table 1 and Supporting Information. The mixture of equal amounts of light and heavy YYC370 were also subjected to our quantitation, and the ratios for each protein levels were ranged from 1.02 to 1.33, indicating the accuracy of our quantitative analysis.

Within 15 CWPs analyzed, cell wall proteins Cis3p, Crh1p, Hsp150p, and Scw10p were all expressed significantly higher in all 4 clinical isolates than in S288c lab strain, and higher levels of Ecm33p, Pir1p, Pst1p, and Scw4p were observed in 3 out of 4 clinical isolates (Fig 3). Scw10p, Pst1p, and Hsp150p were top 3 proteins expressed at least 2 folds higher in clinical isolates than in S288c lab strain. In contrast, the expression levels of Ccw14p were about the same in all the clinical isolates relative to lab strain. We examined the mRNA levels of *HSP150* in S288c and clinical isolates and found that *HSP150* transcripts in clinical isolates were surprisingly slightly reduced (data not shown). We suspected that the increased Hsp150p protein abundance was more likely due to post-transcriptional regulation. Scw10p, one of ASL-CWPs with potential glucanase activity, has been suggested to play a role in conjugation during mating [48]. However, how the Scw10p contributed to cell surface property of clinical isolates is still uncertain. Notably, we observed Pst1p, a GPI-modified protein homologous to Ecm33p, covalently incorporated in cell wall fraction of exponential-phase cells. Interestingly, our quantification results showed that Pst1p expressed at significantly 3 to 8-fold higher levels in clinical isolates than in S288c lab strain (Fig 3A), while Ecm33p expression only increased modestly about 2 folds. The function of Pst1p is still unknown but it was reported to be upregulated by cell wall damage or by cell integrity pathway [49, 50]. Nevertheless, the *C*. *albicans* ortholog of Pst1p is not found, but Ecm33p is required for its virulence [51]. We therefore speculate that Pst1p in *S*. *cerevisiae* might be correlated to strain pathogenicity. The role of Pst1p up-regulation in clinical isolates of *S*. *cerevisiae* still remains to be further investigated. However, there is no direct evidence showing that Scw10p or Pst1p is related to fungal pathogenicity to our knowledge.

**Fig 4:**
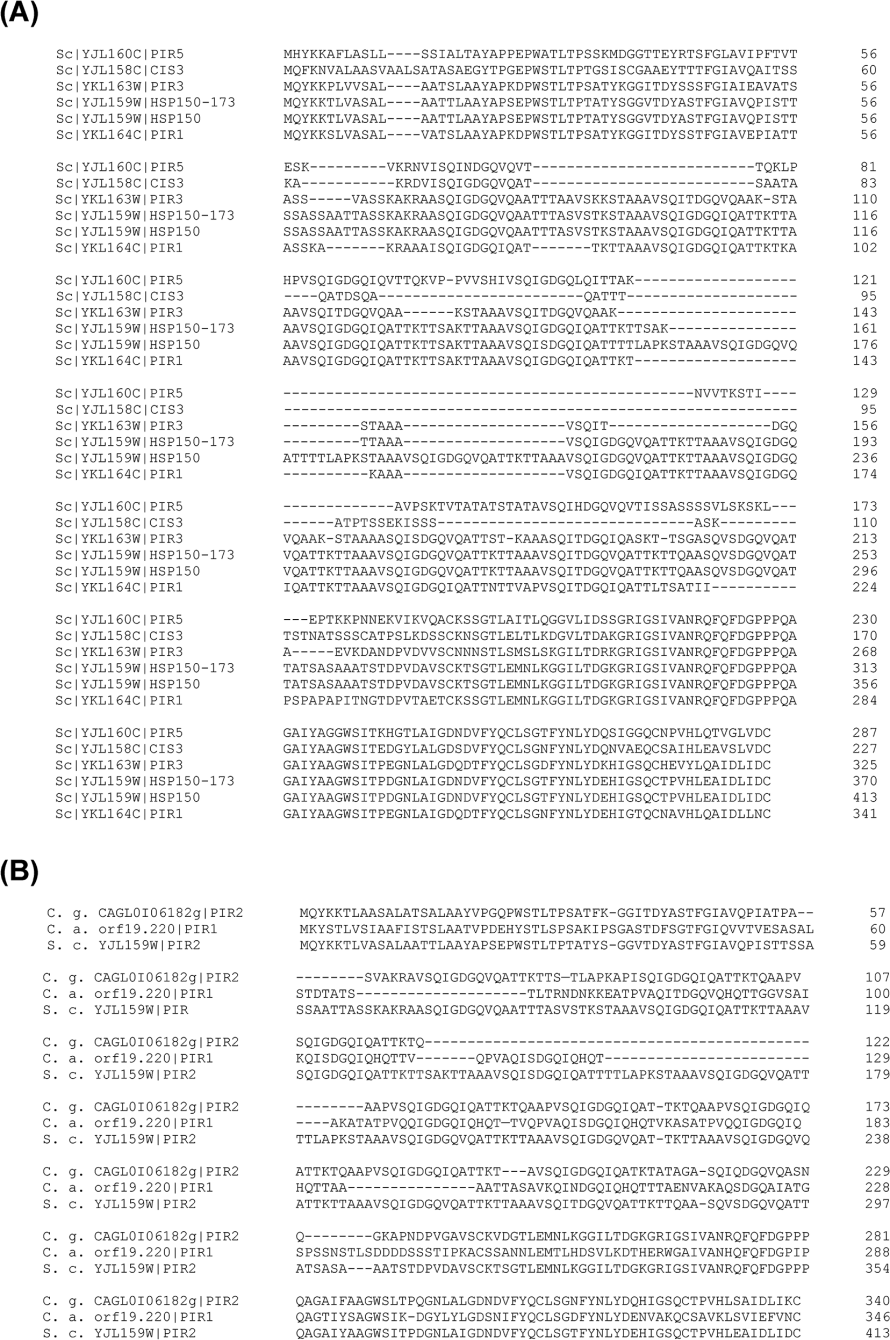
Protein sequence alignment of Pir family. The protein sequence of *HSP150-173*, the *HSP150* allele of clinical isolate YYC173, was deduced from its DNA sequencing results. (A) Pir family protein sequences of Pir1p, Hsp150p, Pir3p, Cis3p, and Pir5p from lab strain S288c, extracted from *Saccharomyces* Genome Database, together with Hsp150-173 were aligned. (B) Hsp150p/Pir2p protein orthologous sequences of *Candida albicans (C*. *a*.*)* and *Candida glabrata (C*. *g*.*)*, extracted from *Candida* Genome Database, as well as sequence of *S*. *cerevisiae* (S. c.) were aligned. All the sequences were aligned by Clustal Omega (http://www.clustal.org/omega/).

Pir proteins, including Pir1p, Pir2/Hsp150p, Pir3p, and Pir4/Cis3p in *S*. *cerevisiae*, apparently attach to cell wall via 1,3-glucan through alkali-sensitive linkages. An N-terminal signal peptide, a Kex2 site, multiple PIR repeat-containing regions, and a carboxyl-terminal region with four cysteine residues were highly conserved in Pir family. Hsp150p/Pir2p is one of the members in Pir family which are regulated by cell integrity pathway and required for cell wall stability [52, 53]. Among 5 Pir proteins found in *S*. *cerevisiae*, despite the fact that Pir5p is still a putative protein of unknown function, and Pir3p was unable to be quantified in our analyses, Pir1p, Hsp150p/Pir2p, and Cis3p/Pir4p levels were all considerably increased in clinical isolates analyzed (Fig 3B). The protein sequence of Hsp150p from clinical isolate YYC173 (Hsp150-173) was aligned with 5 Pir family proteins from lab strain S288c in Fig 4A. Hsp150-173 is one PIR repeat shorter and 98% identical to Hsp150p from S288c lab strain. On the other hand, Hsp150p showed 54–81% identity to other Pir proteins. Amazingly, the C-terminal regions with 4-cysteine domains in particular are highly conserved among all Pir proteins. The sequence data indicated that the Hsp150p protein abundance but not structural difference may play critical roles in the phenotypes we observed in clinical isolates. By comparing Pir2/Hsp150p orthologs across species, *C*. *albicans* ortholog CaPir1p and *C*. *glabrata* ortholog CgPir2p were identified by an *in silico* search of *Candida* Genome Database and aligned in Fig 4B. It showed that Hsp150p/Pir2p orthologs among *S*. *cerevisiae* and *Candida* species are also highly conserved in C-terminal regions including 4 cysteine residues. Surprisingly, the sequence identity of Hsp150p/Pir2p between *S*. *cerevisiae* and *C*. *glabrata* are also as high as 80%. The C-terminal repeated regions are serine/threonine rich, and are thought to be greatly O-mannosylated. In addition, in our quantitative results, other Pir family proteins such as Pir1p and Cis3p were also expressed at moderate higher levels in the clinical isolates, suggesting that the abundance of Pir proteins might play a certain role exceptionally in clinical isolates.

Kex2p is a calcium-dependent serine protease, and Kex2 sites were conserved in all Pir proteins of *S*. *cerevisiae*. It has been reported that CaKex2p is required for hyphal growth and virulence in mice [54], suggesting that *S*. *cerevisiae* Pir proteins, the Kex2p substrates, might also play roles in fungal virulence. CaPir1p with PIR repeats is essential in *C*. *albicans* [55]. CaPir1p showed 9 PIR repeats, whereas we observed ScPir1p, ScPir2/Hsp150p, ScPir3p, and ScPir4p with 8, 10, 7, and 1 repeat(s) respectively. Whether the number of repeats play a role in *S*. *cerevisiae* is still unclear yet.

### High Levels of Hsp150p Enhance Cell Wall Integrity and the Ability of Adherence to Polystyrene Surface

**Fig 5:**
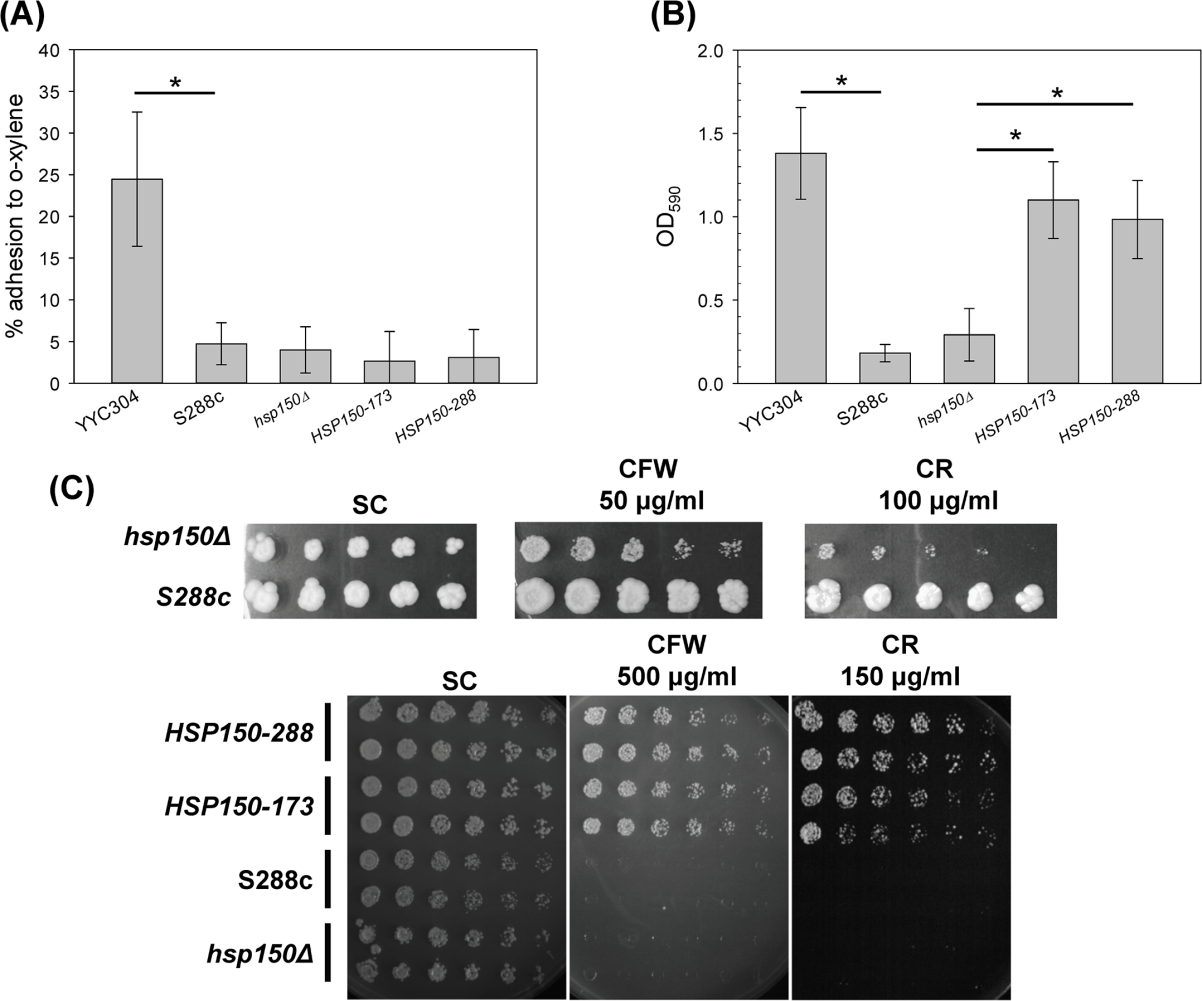
Overexpression of Hsp150p increased cell wall integrity and the ability of adherence to polystyrene surface. Strains S288c and YYC304 were served as negative and positive controls respectively. The *hsp150Δ* strain containing the control vector, the allele *HSP150-288* of S288c strain, or the allele *HSP150-173* of YYC173, were subjected to cell hydrophobicity assay (A), adhesion assay (B), and sensitivity assay with CFW or CR with indicated concentrations (C) as described. Serial 2-fold dilutions of each strain were spotted on solid SC-galactose media or in the presence of CFW or CR. The plates were incubated at 30°C for 3 days. 3 biological replicates were performed. Asterisks indicate statistically significant differences (*p* < 0.05).

In order to further investigate whether higher levels of Hsp150p play roles in cell wall property, we tested the cell hydrophobicity and plastic adhesion ability in *HSP150* overexpressed S288c lab strain. We found that cell hydrophobicity was not significantly changed in *hsp150*-deleted strain or *HSP150* overexpressed strains (Fig 5A), indicating that Hsp150p expression perhaps showed modest effect on cell surface hydrophobicity. On the other hand, plastic adhesion increased up to 3 to 4 folds in *HSP150* overexpressed strains (Fig 5B), suggesting that Hsp150p may be important for the ability of adherence to polystyrene surface. We also investigated the property of cell wall structure and integrity in *HSP150* overexpressed cells with the treatment of higher concentration of CR (150 μg/ml) or CFW (500 μg/ml). Although *hsp150Δ* was still more sensitive than WT at low concentrations of CR (100 μg/ml) or CFW (50 μg/ml), our data revealed that at higher concentrations of CR or CFW, strains with highly expressed Hsp150p were much more resistant to cell wall-perturbing agents, while the WT and *hsp150Δ* were very sensitive (Fig 5C). Deletion of *HSP150* in YYC1 background also increased the sensitivity to CR or CFW. It suggested that Hsp150p played a crucial role in cell wall integrity. All our data suggested that up-regulation of cell wall protein Hsp150p is essential for maintenance of cell wall characteristics in yeasts.

### Overexpression of Hsp150p Enhances the Virulence in Yeast

**Fig 6:**
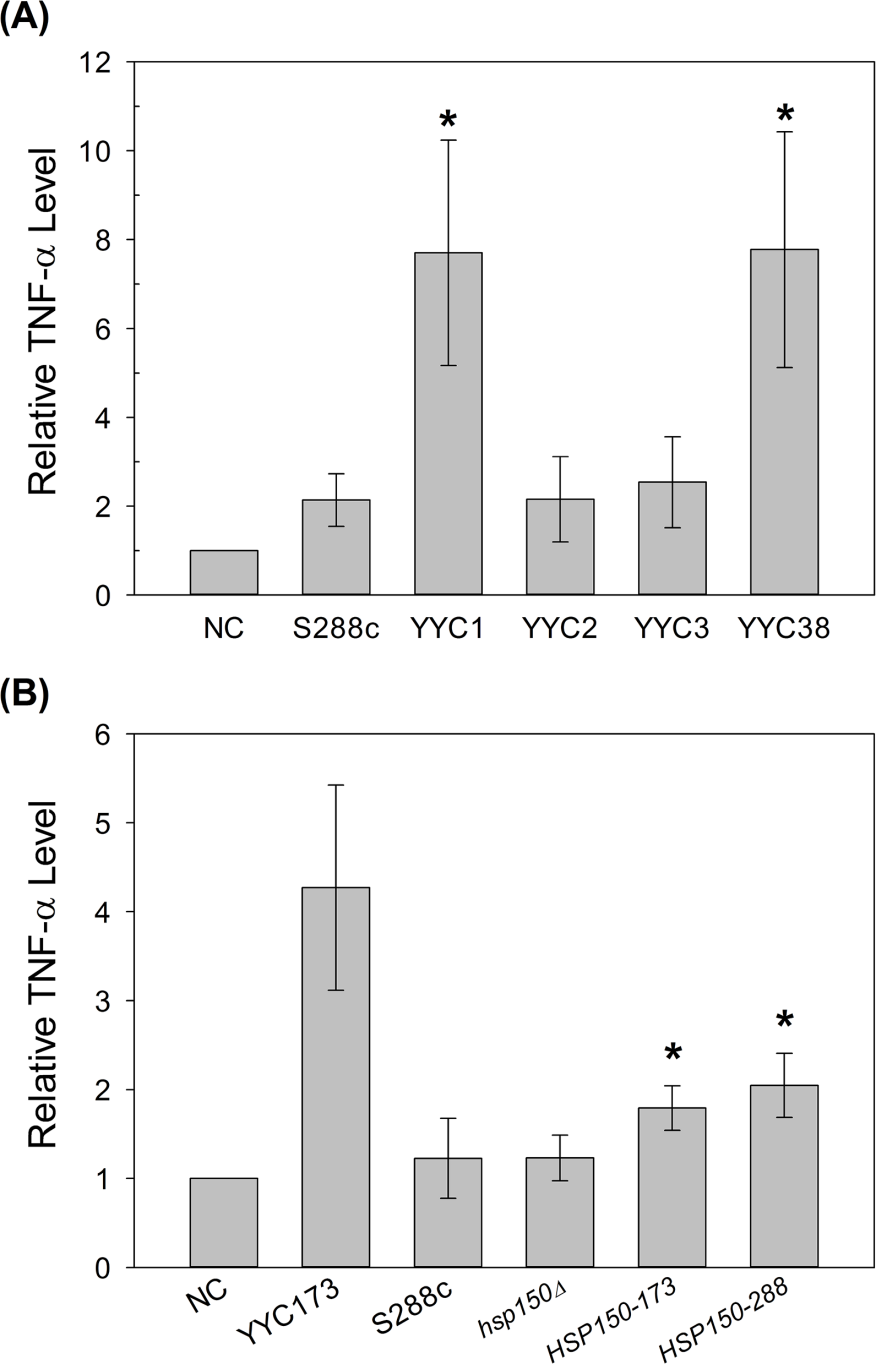
Overexpression of Hsp150p elicits greater proinflammatory cytokine TNF-α induction in macrophage cell line. Murine macrophage cell line RAW264.7 was exposed to clinical isolates (A), and YYC173, *hsp150Δ* strain containing the control vector, overexpressed the allele *HSP150-288*, or allele *HSP150-173* (B) as indicated. NC, macrophage treated without any yeast cells. 2 or 3 biological replicates were performed. Asterisks indicate statistically significant differences from S288c (A) or *hsp150Δ* (B) (*p* < 0.05).

Yeast cell wall is the outermost structure which potentially leads to misrecognition and overstimulation of proinflammatory cytokines by host innate immune system. We further tested whether the changes of cell wall protein composition may be one of the virulence traits in the clinical isolates we observed. The enhancement of TNF-α production stimulated by lab strains and clinical isolates was performed in murine macrophage cell line RAW264.7. We found that comparison to the WT lab strain, clinical isolates YYC1 and YYC38 stimulated at least 3-fold more TNF-α (Fig 6A). Overexpression of Hsp150p in lab strains also showed the enhancement of TNF-α production; however, the induction levels showed no difference between *HSP150-288* and *HSP150-173* alleles (Fig 6B). Our results indicated that the hypervirulence of clinical isolates may be, at least partially, due to the proinflammatory response overstimulated by overexpression of certain cell wall proteins, for example Hsp150p. Moreover, we speculated that Pir2/Hsp150p might be the one in Pir family that is the most functionally equivalent to CaPir1p in fungal pathogenicity.

Fungal virulence was considered multi-factorial. Several key virulence factors have been addressed in *C*. *albicans*. Secreted aspartic proteases (Saps) have been recognized as a virulence-associated trait [56, 57]. The infectious ability of *C*. *albicans* is closely related to its ability of biofilm formation [58, 59]. In *Aspergillus fumigatus*, a protein phosphatase 2A SitA was identified as a possible modulator for adhesion, cell wall integrity, biofilm formation, and virulence [60]. Several virulence factors have been addressed in *S*. *cerevisiae* such as thermotolerance and pseudohyphal formation [61]. Although the emergence of virulent isolates is still puzzling, here we reported that the change in cell wall protein composition in *S*. *cerevisiae* is related to its hydrophobicity, adhesion, cell wall integrity, and virulence.

## Conclusions

The yeast cell wall is a dynamic structure with a layer of glucan and chitin molecules as scaffold for the outer layer of glycoproteins, and we reported here the first study of cell wall quantitative proteomics in clinical isolates of *S*. *cerevisiae* by SILAC labeling followed by LC-MS/MS analysis. Our results indicated that clinical isolates of *S*. *cerevisiae* showed distinct characteristics of cell wall, such as higher hydrophobicity, higher ability of adherence, and higher levels of certain CWPs, including Hsp150p, indicating the discrepancy of the cell wall structure or composition between clinical isolates and lab strain S288c. In addition, we also showed that overexpression of Hsp150p enhances the virulence of yeast. Our findings in this study will lead to a better understanding of CWPs as well as the pathogenicity of clinical isolates of *S*. *cerevisiae*.

## Supporting Information

S1 Fig An illustration of the mass shift and quantification for peptide VGQSLSIVSNDELSK with Lys-D0 and Lys-D4 labeling. (A) MS spectrum shows a 2 m/z difference for doubly charged peptides; and (B) MS/MS spectrum shows no different for b11, b12, and b13 ions but with a 4 m/z shift for y11 and y12 ions. (C) Manual quantification of peaks for peptides with both Lys-D0 and Lys-D4 labeling from LC chromatogram.
(PDF)

S1 Table List of peptides from cell wall proteins for SILAC quantification. (PDF)

S2 Table List of peptides from cytoplasmic proteins as the internal control for SILAC quantification. (PDF)

S3 Table The averaged relative quantitative levels of cell wall proteins analyzed by MassMatrix (MM) and MaxQuant (MQ). (PDF)

## References

[1] Arendrup MC, Sulim S, Holm A, Nielsen L, Nielsen SD, Knudsen JD, et al Diagnostic issues, clinical characteristics, and outcomes for patients with fungemia. J Clin Microbiol. 2011;49(9):3300–8. 10.1128/JCM.00179-11 21715585 PMC3165619

[2] Aucott JN, Fayen J, Grossnicklas H, Morrissey A, Lederman MM, Salata RA. Invasive infection with Saccharomyces cerevisiae: report of three cases and review. Reviews of infectious diseases. 1990;12(3):406–11. . 2193348 10.1093/clinids/12.3.406

[3] Chitasombat MN, Kofteridis DP, Jiang Y, Tarrand J, Lewis RE, Kontoyiannis DP. Rare opportunistic (non-Candida, non-Cryptococcus) yeast bloodstream infections in patients with cancer. The Journal of infection. 2012;64(1):68–75. 10.1016/j.jinf.2011.11.002 22101079 PMC3855381

[4] Cimolai N, Gill MJ, Church D. Saccharomyces cerevisiae fungemia: case report and review of the literature. Diagn Microbiol Infect Dis. 1987;8(2):113–7. . 3322656 10.1016/0732-8893(87)90158-1

[5] Kiehn TE, Edwards FF, Armstrong D. The prevalence of yeasts in clinical specimens from cancer patients. American journal of clinical pathology. 1980;73(4):518–21. . 7369176 10.1093/ajcp/73.4.518

[6] Tawfik OW, Papasian CJ, Dixon AY, Potter LM. Saccharomyces cerevisiae pneumonia in a patient with acquired immune deficiency syndrome. J Clin Microbiol. 1989;27(7):1689–91. 2671026 10.1128/jcm.27.7.1689-1691.1989 PMC267645

[7] McCusker JH, Clemons KV, Stevens DA, Davis RW. Genetic characterization of pathogenic Saccharomyces cerevisiae isolates. Genetics. 1994;136(4):1261–9. 8013903 10.1093/genetics/136.4.1261 PMC1205906

[8] Byron JK, Clemons KV, McCusker JH, Davis RW, Stevens DA. Pathogenicity of Saccharomyces cerevisiae in complement factor five-deficient mice. Infect Immun. 1995;63(2):478–85. Epub 1995/02/01. . 7822013 10.1128/iai.63.2.478-485.1995 PMC173020

[9] Clemons KV, McCusker JH, Davis RW, Stevens DA. Comparative pathogenesis of clinical and nonclinical isolates of Saccharomyces cerevisiae. J Infect Dis. 1994;169(4):859–67. Epub 1994/04/01. . 8133102 10.1093/infdis/169.4.859

[10] Hazen KC. New and emerging yeast pathogens. Clinical microbiology reviews. 1995;8(4):462–78. 8665465 10.1128/cmr.8.4.462 PMC172871

[11] Miceli MH, Diaz JA, Lee SA. Emerging opportunistic yeast infections. The Lancet Infectious diseases. 2011;11(2):142–51. 10.1016/S1473-3099(10)70218-8 . 21272794

[12] Muller LA, Lucas JE, Georgianna DR, McCusker JH. Genome-wide association analysis of clinical vs. nonclinical origin provides insights into Saccharomyces cerevisiae pathogenesis. Molecular ecology. 2011;20(19):4085–97. 10.1111/j.1365-294X.2011.05225.x 21880084 PMC3183415

[13] Murphy JW. Immunological down-regulation of host defenses in fungal infections. Mycoses. 1999;42 Suppl 2:37–43. . 10865902

[14] Ponton J, Ruchel R, Clemons KV, Coleman DC, Grillot R, Guarro J, et al Emerging pathogens. Medical mycology. 2000;38 Suppl 1:225–36. . 11204149 10.1080/mmy.38.s1.225.236

[15] Janeway CA Jr., Medzhitov R. Innate immune recognition. Annual review of immunology. 2002;20:197–216. 10.1146/annurev.immunol.20.083001.084359 . 11861602

[16] Underhill DM, Ozinsky A. Phagocytosis of microbes: complexity in action. Annual review of immunology. 2002;20:825–52. 10.1146/annurev.immunol.20.103001.114744 . 11861619

[17] Sutherland IW. Novel and established applications of microbial polysaccharides. Trends in biotechnology. 1998;16(1):41–6. 10.1016/S0167-7799(97)01139-6 . 9470230

[18] Chaffin WL, Lopez-Ribot JL, Casanova M, Gozalbo D, Martinez JP. Cell wall and secreted proteins of Candida albicans: identification, function, and expression. Microbiol Mol Biol Rev. 1998;62(1):130–80. Epub 1998/04/08. . 9529890 10.1128/mmbr.62.1.130-180.1998 PMC98909

[19] Garcia-Sanchez S, Aubert S, Iraqui I, Janbon G, Ghigo JM, d'Enfert C. Candida albicans biofilms: a developmental state associated with specific and stable gene expression patterns. Eukaryotic cell. 2004;3(2):536–45. Epub 2004/04/13. . 15075282 10.1128/EC.3.2.536-545.2004 PMC387656

[20] Hoyer LL. The ALS gene family of Candida albicans. Trends Microbiol. 2001;9(4):176–80. Epub 2001/04/05. doi: S0966-842X(01)01984-9 [pii]. . 11286882 10.1016/s0966-842x(01)01984-9

[21] Klis FM, Mol P, Hellingwerf K, Brul S. Dynamics of cell wall structure in Saccharomyces cerevisiae. FEMS Microbiol Rev. 2002;26(3):239–56. Epub 2002/08/08. doi: S0168644502000876 [pii]. . 12165426 10.1111/j.1574-6976.2002.tb00613.x

[22] Klotz SA, Gaur NK, Lake DF, Chan V, Rauceo J, Lipke PN. Degenerate peptide recognition by Candida albicans adhesins Als5p and Als1p. Infect Immun. 2004;72(4):2029–34. Epub 2004/03/25. . 15039323 10.1128/IAI.72.4.2029-2034.2004 PMC375204

[23] Sundstrom P. Adhesion in Candida spp. Cell Microbiol. 2002;4(8):461–9. Epub 2002/08/14. doi: 206 [pii]. . 12174081 10.1046/j.1462-5822.2002.00206.x

[24] Brown GD, Taylor PR, Reid DM, Willment JA, Williams DL, Martinez-Pomares L, et al Dectin-1 is a major beta-glucan receptor on macrophages. The Journal of experimental medicine. 2002;196(3):407–12. 12163569 10.1084/jem.20020470 PMC2193936

[25] Mansour MK, Levitz SM. Interactions of fungi with phagocytes. Curr Opin Microbiol. 2002;5(4):359–65. . 12160853 10.1016/s1369-5274(02)00342-9

[26] Wheeler RT, Kupiec M, Magnelli P, Abeijon C, Fink GR. A Saccharomyces cerevisiae mutant with increased virulence. Proceedings of the National Academy of Sciences of the United States of America. 2003;100(5):2766–70. 10.1073/pnas.0437995100 12589024 PMC151415

[27] Astiz ME, Rackow EC. Septic shock. Lancet. 1998;351(9114):1501–5. 10.1016/S0140-6736(98)01134-9 . 9605819

[28] Klis FM, de Groot P, Hellingwerf K. Molecular organization of the cell wall of Candida albicans. Medical mycology. 2001;39 Suppl 1:1–8. . 11800263

[29] Weig M, Jansch L, Gross U, De Koster CG, Klis FM, De Groot PW. Systematic identification in silico of covalently bound cell wall proteins and analysis of protein-polysaccharide linkages of the human pathogen Candida glabrata. Microbiology. 2004;150(Pt 10):3129–44. 10.1099/mic.0.27256-0 . 15470094

[30] de Godoy LM, Olsen JV, de Souza GA, Li G, Mortensen P, Mann M. Status of complete proteome analysis by mass spectrometry: SILAC labeled yeast as a model system. Genome biology. 2006;7(6):R50 10.1186/gb-2006-7-6-r50 16784548 PMC1779535

[31] Ong SE, Blagoev B, Kratchmarova I, Kristensen DB, Steen H, Pandey A, et al Stable isotope labeling by amino acids in cell culture, SILAC, as a simple and accurate approach to expression proteomics. Mol Cell Proteomics. 2002;1(5):376–86. . 12118079 10.1074/mcp.m200025-mcp200

[32] Ong SE, Mann M. Mass spectrometry-based proteomics turns quantitative. Nature chemical biology. 2005;1(5):252–62. 10.1038/nchembio736 . 16408053

[33] Kovacs M, Stuparevic I, Mrsa V, Maraz A. Characterization of Ccw7p cell wall proteins and the encoding genes of Saccharomyces cerevisiae wine yeast strains: relevance for flor formation. FEMS yeast research. 2008;8(7):1115–26. 10.1111/j.1567-1364.2008.00413.x . 18657192

[34] Peeters E, Nelis HJ, Coenye T. Comparison of multiple methods for quantification of microbial biofilms grown in microtiter plates. Journal of microbiological methods. 2008;72(2):157–65. 10.1016/j.mimet.2007.11.010 . 18155789

[35] Reynolds TB, Fink GR. Bakers' yeast, a model for fungal biofilm formation. Science. 2001;291(5505):878–81. 10.1126/science.291.5505.878 . 11157168

[36] de Groot PW, de Boer AD, Cunningham J, Dekker HL, de Jong L, Hellingwerf KJ, et al Proteomic analysis of Candida albicans cell walls reveals covalently bound carbohydrate-active enzymes and adhesins. Eukaryotic cell. 2004;3(4):955–65. 10.1128/EC.3.4.955-965.2004 15302828 PMC500891

[37] Yin QY, de Groot PW, Dekker HL, de Jong L, Klis FM, de Koster CG. Comprehensive proteomic analysis of Saccharomyces cerevisiae cell walls: identification of proteins covalently attached via glycosylphosphatidylinositol remnants or mild alkali-sensitive linkages. The Journal of biological chemistry. 2005;280(21):20894–901. 10.1074/jbc.M500334200 . 15781460

[38] Xu H, Freitas MA. MassMatrix: a database search program for rapid characterization of proteins and peptides from tandem mass spectrometry data. Proteomics. 2009;9(6):1548–55. 10.1002/pmic.200700322 19235167 PMC2759086

[39] Xu H, Hsu PH, Zhang L, Tsai MD, Freitas MA. Database search algorithm for identification of intact cross-links in proteins and peptides using tandem mass spectrometry. Journal of proteome research. 2010;9(7):3384–93. 10.1021/pr100369y 20469931 PMC4141472

[40] Lin JS, Huang JH, Hung LY, Wu SY, Wu-Hsieh BA. Distinct roles of complement receptor 3, Dectin-1, and sialic acids in murine macrophage interaction with Histoplasma yeast. J Leukoc Biol. 2010;88(1):95–106. 10.1189/jlb.1109717 . 20360401

[41] Nakamura LT, Wu-Hsieh BA, Howard DH. Recombinant murine gamma interferon stimulates macrophages of the RAW cell line to inhibit intracellular growth of Histoplasma capsulatum. Infect Immun. 1994;62(2):680–4. 8300224 10.1128/iai.62.2.680-684.1994 PMC186157

[42] Galan-Ladero MA, Blanco-Blanco MT, Hurtado C, Perez-Giraldo C, Blanco MT, Gomez-Garcia AC. Determination of biofilm production by Candida tropicalis isolated from hospitalized patients and its relation to cellular surface hydrophobicity, plastic adherence and filamentation ability. Yeast. 2013;30(9):331–9. 10.1002/yea.2965 . 23775541

[43] van der Mei HC, de Vries J, Busscher HJ. Hydrophobic and electrostatic cell surface properties of thermophilic dairy streptococci. Applied and environmental microbiology. 1993;59(12):4305–12. 16349127 10.1128/aem.59.12.4305-4312.1993 PMC195901

[44] Mrsa V, Klebl F, Tanner W. Purification and characterization of the Saccharomyces cerevisiae BGL2 gene product, a cell wall endo-beta-1,3-glucanase. Journal of bacteriology. 1993;175(7):2102–6. 8458852 10.1128/jb.175.7.2102-2106.1993 PMC204315

[45] Plotnikova TA, Selyakh IO, Kalebina TS, Kulaev IS. Bgl2p and Gas1p are the major glucan transferases forming the molecular ensemble of yeast cell wall. Doklady Biochemistry and biophysics. 2006;409:244–7. . 16986442 10.1134/s1607672906040144

[46] Lopez-Ribot JL, Chaffin WL. Members of the Hsp70 family of proteins in the cell wall of Saccharomyces cerevisiae. Journal of bacteriology. 1996;178(15):4724–6. 8755907 10.1128/jb.178.15.4724-4726.1996 PMC178246

[47] Delgado ML, O'Connor JE, Azorin I, Renau-Piqueras J, Gil ML, Gozalbo D. The glyceraldehyde-3-phosphate dehydrogenase polypeptides encoded by the Saccharomyces cerevisiae TDH1, TDH2 and TDH3 genes are also cell wall proteins. Microbiology. 2001;147(Pt 2):411–7. . 11158358 10.1099/00221287-147-2-411

[48] Cappellaro C, Mrsa V, Tanner W. New potential cell wall glucanases of Saccharomyces cerevisiae and their involvement in mating. Journal of bacteriology. 1998;180(19):5030–7. 9748433 10.1128/jb.180.19.5030-5037.1998 PMC107536

[49] Jung US, Levin DE. Genome-wide analysis of gene expression regulated by the yeast cell wall integrity signalling pathway. Molecular microbiology. 1999;34(5):1049–57. . 10594829 10.1046/j.1365-2958.1999.01667.x

[50] Terashima H, Yabuki N, Arisawa M, Hamada K, Kitada K. Up-regulation of genes encoding glycosylphosphatidylinositol (GPI)-attached proteins in response to cell wall damage caused by disruption of FKS1 in Saccharomyces cerevisiae. Molecular & general genetics: MGG. 2000;264(1–2):64–74. . 11016834 10.1007/s004380000285

[51] Martinez-Lopez R, Monteoliva L, Diez-Orejas R, Nombela C, Gil C. The GPI-anchored protein CaEcm33p is required for cell wall integrity, morphogenesis and virulence in Candida albicans. Microbiology. 2004;150(Pt 10):3341–54. 10.1099/mic.0.27320-0 . 15470113

[52] Kapteyn JC, Van Egmond P, Sievi E, Van Den Ende H, Makarow M, Klis FM. The contribution of the O-glycosylated protein Pir2p/Hsp150 to the construction of the yeast cell wall in wild-type cells and beta 1,6-glucan-deficient mutants. Molecular microbiology. 1999;31(6):1835–44. . 10209754 10.1046/j.1365-2958.1999.01320.x

[53] Mrsa V, Tanner W. Role of NaOH-extractable cell wall proteins Ccw5p, Ccw6p, Ccw7p and Ccw8p (members of the Pir protein family) in stability of the Saccharomyces cerevisiae cell wall. Yeast. 1999;15(10A):813–20. 10.1002/(SICI)1097-0061(199907)15:10A<813::AID-YEA421>3.0.CO;2-Y . 10407261

[54] Newport G, Kuo A, Flattery A, Gill C, Blake JJ, Kurtz MB, et al Inactivation of Kex2p diminishes the virulence of Candida albicans. The Journal of biological chemistry. 2003;278(3):1713–20. 10.1074/jbc.M209713200 . 12419804

[55] Martinez AI, Castillo L, Garcera A, Elorza MV, Valentin E, Sentandreu R. Role of Pir1 in the construction of the Candida albicans cell wall. Microbiology. 2004;150(Pt 10):3151–61. 10.1099/mic.0.27220-0 . 15470096

[56] Naglik JR, Challacombe SJ, Hube B. Candida albicans secreted aspartyl proteinases in virulence and pathogenesis. Microbiol Mol Biol Rev. 2003;67(3):400–28, table of contents. 12966142 10.1128/MMBR.67.3.400-428.2003 PMC193873

[57] Naglik JR, Moyes D, Makwana J, Kanzaria P, Tsichlaki E, Weindl G, et al Quantitative expression of the Candida albicans secreted aspartyl proteinase gene family in human oral and vaginal candidiasis. Microbiology. 2008;154(Pt 11):3266–80. 10.1099/mic.0.2008/022293-0 18957581 PMC2722715

[58] Douglas LJ. Candida biofilms and their role in infection. Trends Microbiol. 2003;11(1):30–6. . 12526852 10.1016/s0966-842x(02)00002-1

[59] Nobile CJ, Mitchell AP. Genetics and genomics of Candida albicans biofilm formation. Cell Microbiol. 2006;8(9):1382–91. 10.1111/j.1462-5822.2006.00761.x . 16848788

[60] Bom VL, de Castro PA, Winkelstroter LK, Marine M, Hori JI, Ramalho LN, et al Aspergillus fumigatus sitA phosphatase homologue is important for adhesion, cell wall integrity, biofilm formation and virulence. Eukaryotic cell. 2015 10.1128/EC.00008-15 . 25911225 PMC4519751

[61] McCusker JH, Clemons KV, Stevens DA, Davis RW. Saccharomyces cerevisiae virulence phenotype as determined with CD-1 mice is associated with the ability to grow at 42 degrees C and form pseudohyphae. Infect Immun. 1994;62(12):5447–55. Epub 1994/12/01. . 7960125 10.1128/iai.62.12.5447-5455.1994 PMC303287

