# Supplementary material for: Characterization of Cell Wall Proteins in Saccharomyces cerevisiae Clinical Isolates Elucidates Hsp150p in Virulence
Source: PLoS One. 2015 Aug 13;10(8):e0135174. doi: 10.1371/journal.pone.0135174 (PMC4535956; doi:10.1371/journal.pone.0135174)
Supplement: S1 Fig — (A) MS spectrum shows a 2 m/z difference for doubly charged peptides; and (B) MS/MS spectrum shows no different for b11, b12, and b13 ions but with a 4 m/z shift for y11 and y12 ions. (C) Manual quantification of peaks for peptides with both Lys-D0 and Lys-D4 labeling from LC chromatogram. (PDF) [file pone.0135174.s001.pdf]

S1 Figure: **An illustration of the mass shift and quantification for peptide VGQSLSIVSNDELSK with Lys-D0 and Lys-D4 labeling.** (A) MS spectrum shows a 2 m/z difference for doubly charged peptides; and (B) MS/MS spectrum shows no different for b_{11}, b_{12}, and b_{13} ions but with a 4 m/z shift for y_{11} and y_{12} ions. (C) Manual quantification of peaks for peptides with both Lys-D0 and Lys-D4 labeling from LC chromatogram.

(A)

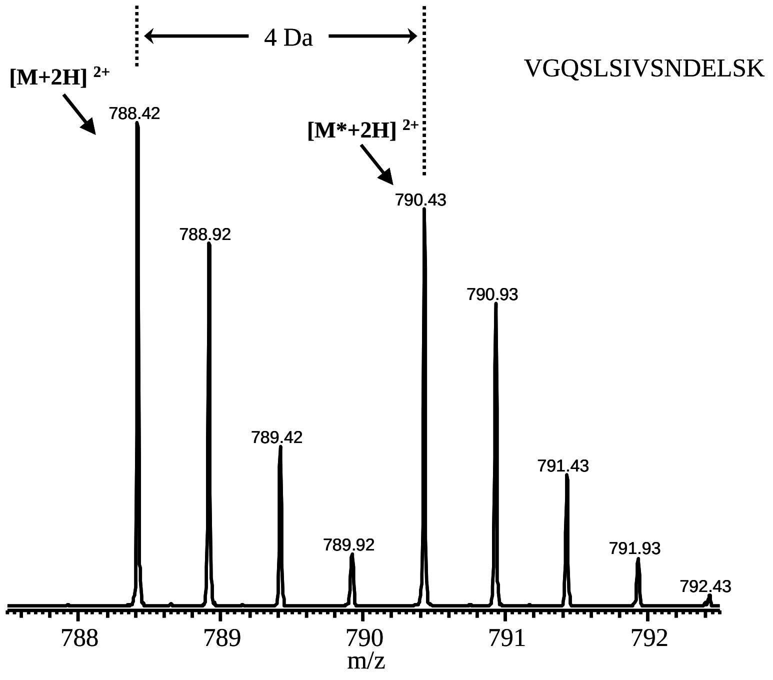

(B)

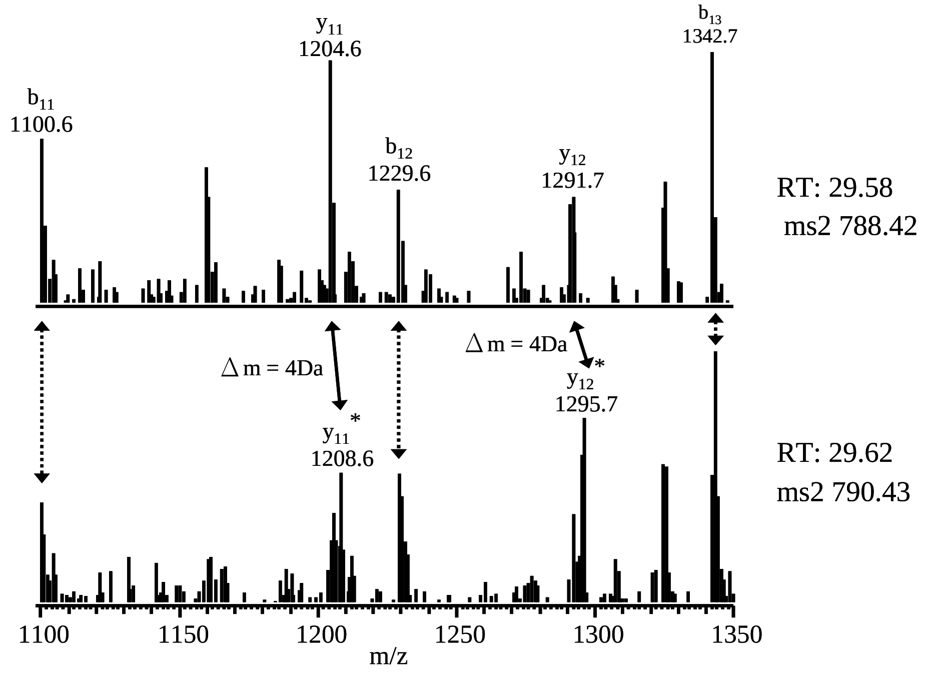
b11
1100.6
y11
1204.6
b12
1229.6
y12
1291.7
b13
1342.7
RT: 29.58
ms2 788.42
Δ m = 4Da
Δ m = 4Da
y12 *
1295.7
y11 *
1208.6
RT: 29.62
ms2 790.43
1100
1150
1200
1250
1300
1350
m/z

(C)

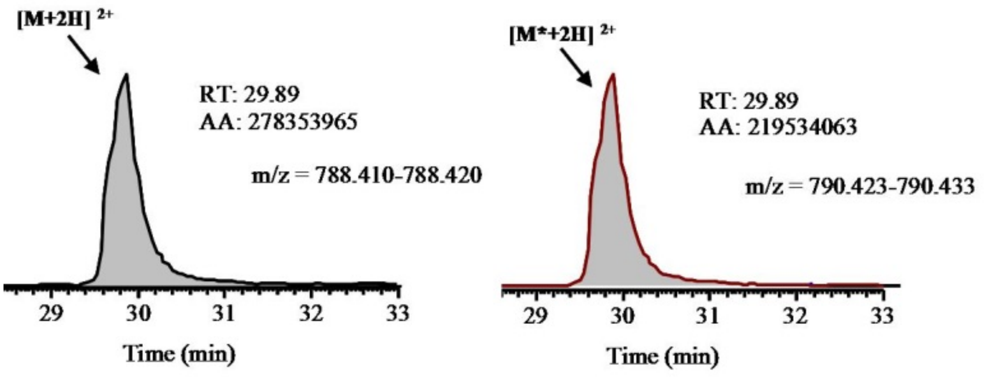
[M+2H] 2+
RT: 29.89
AA: 278353965
m/z = 788.410-788.420
29
30
31
32
33
Time (min)
[M*+2H] 2+
RT: 29.89
AA: 219534063
m/z = 790.423-790.433
29
30
31
32
33
Time (min)
