# Supplementary material for: Characterization of Cell Wall Proteins in Saccharomyces cerevisiae Clinical Isolates Elucidates Hsp150p in Virulence
Source: PLoS One. 2015 Aug 13;10(8):e0135174. doi: 10.1371/journal.pone.0135174 (PMC4535956; doi:10.1371/journal.pone.0135174)
Supplement: S1 Table — (PDF) [file pone.0135174.s002.pdf]

SUPPORTING INFORMATION.

S1 Table: List of peptides from cell wall proteins for SILAC quantification.

Protein ID	Sequence		$[M+H]^+$	Peptide Sequence[b, c, d]
	Start	End		
Bgl2p	111	128	1940.05	IKESTVAGFLVGSEALYR
Crh1p	**68**	**73**	**809.42**	**WFTDLK**
	**74**	**79**	**653.35**	**HAGEIK**
	**74**	**91**	**1877.94**	**HAGEIKYGSDGLSMTLAK**
	**80**	**91**	**1242.60**	**YGSDGLSMTLAK**
	**92**	**99**	**992.52**	**RYDNPSLK**
	**93**	**99**	**836.42**	**YDNPSLK**
	**100**	**108**	**1122.53**	**SNFYIMYGK**
	**109**	**114**	**714.48**	**LEVILK**
	187	197	1326.63	TTWYLDGESVR
	252	261	1082.57	VIVTDYSTGK
	**252**	**262**	**1210.67**	**VIVTDYSTGKK**
	263	285	2498.07	YTYGDQSGSWESIEADGGSIYGR
Crh2p	**60**	**74**	1827.79	**YSFSHDSCMPVPICK**
	**80**	**96**	**874.43**	**FKDYSSK**
	124	134	1078.55	NSGGTVLSSTR
Cwp1p	**32**	**49**	**1946.93**	**SGSDLQYLSVYSDNGTLK**
	**50**	**66**	**1641.76**	**LGSGSGSFEATITDDGK**
	**50**	**68**	**1882.94**	**LGSGSGSFEATITDDGKLK**
	**106**	**114**	**959.48**	**SSSGFYAIK**
	**106**	**124**	**2030.97**	**SSSGFYAIKDGSSYIFSSK**
	**115**	**124**	**1090.51**	**DGSSYIFSSK**
	**125**	**139**	**1545.79**	**QSDDATGVAIRPTSK**
Ccw12p	117	133	1476.91	ALPAAGALLAGAAALLL
Ccw14p	**38**	**60**	**2676.10**	**SSSTCDSLNQVTCYCEHENSAVK**

	**38**	**61**	**2804.19**	**SSSTCDSLNQVTCYCEHENSAVKK**
	**61**	**80**	**2260.00**	**KCLDSICPNNDADAAYSAFK**
	**62**	**80**	**2131.91**	**CLDSICPNNDADAAYSAFK**
Cwp2p	76	92	1458.78	AAVGMGAGALAAAAMLL
Ecm33p	**162**	**172**	**1331.72**	**KVNVFNINNNR**
	**163**	**172**	**1203.62**	**VNVFNINNNR**
	**248**	**262**	**1575.82**	**VGQSLSIVSNDELSK**
	**285**	**291**	**792.43**	**VIDGFNK**
	**337**	**351**	**1623.78**	**LQSNGAIQGDSFVCK**
Exg2p	208	218	1205.67	ILYGDLGWLR
Gas1p	**83**	**90**	**957.58**	**KLNTNVIR**
	106	127	2287.19	ALNDADIYVIADLAAPATSINR
	**128**	**141**	**1700.78**	**DDPTWTVDLFNSYK**
	**184**	**192**	**1186.59**	**QYISDKNYR**
	**193**	**207**	**1695.78**	**KIPVGYSSNDDEDTR**
	**193**	**209**	**1922.95**	**KIPVGYSSNDDEDTRVK**
	194	207	1567.69	IPVGYSSNDDEDTR
	210	222	1536.60	MTDYFACGDDDVK
	**223**	**236**	**1753.74**	**ADFYGINMYEWCGK**
	241	247	769.35	TSGYADR
	**415**	**424**	**1193.53**	**YGAYSFCTPK**
Gas3p	**32**	**51**	**2246.10**	**FIKPSSATNSESDNEVFFVK**
	52	80	3046.29	GVDYQPGGSSGYDADSDTDILSDPEVCAR
	81	94	1581.80	DAYAFQQLGVNTVR
	134	147	1544.72	ADPSGTYDSLYLSR
	203	217	1578.78	SIPVGYSAADNTDLR
	223	242	2311.09	YLQCNSLDGNKVNDDLDISK
	**293**	**306**	**1514.74**	**TFDEVSEGLYGGLK**
	**329**	**349**	**2387.16**	**LDDSGSLTYKDDFVNLESQLK**
	**358**	**368**	**1257.59**	**ESEISSDSIYK**
Gas5p	34	45	1314.57	GNAFFNSESGER
	192	206	1573.83	QIPVGYSAADIVANR
	207	222	1829.78	QLAAEYFNCGDEADAR
	**248**	**269**	**2652.28**	**MKLYQDYSVPVFLSEFGCNQVK**
	**250**	**269**	**2393.15**	**LYQDYSVPVFLSEFGCNQVK**

	**317**	**329**	**1600.79**	**LTDFENLKNEYSK**
Mkc7p	305	321	1988.06	YAGDLYTIPIINTLQHR
Pry3	**121**	**130**	**1085.49**	**STAEIGCGYK**
Plb2p	101	111	1110.54	IGIACSGGGYR
Pst1p	**126**	**140**	**1604.93**	**LITLPAISSFTSNIK**
	246	262	1913.00	LNTIGQTFSIVSNDYLK
	319	326	762.36	GGADVESK
Pir1p	**246**	**255**	**1079.54**	**SSGTLEMNLK**
	246	263	1820.94	SSGTLEMNLKGGILTDGK
	**246**	**265**	**2034.07**	**SSGTLEMNLKGGILTDGKGR**
Pir2p	**318**	**327**	**1093.56**	**TSGTLEMNLK**
	318	335	1834.96	TSGTLEMNLKGGILTDGK
	**318**	**337**	**2048.08**	**TSGTLEMNLKGGILTDGKGR**
Pir3p	307	325	2271.05	HIGSQCHEVYLQAIDLIDC
Cis3p	**127**	**149**	**2452.22**	**DSSCKNSGTLELTLKDGVLTDAK**
	127	151	2665.35	DSSCKNSGTLELTLKDGVLTDAKGR
	**132**	**141**	**1075.60**	**NSGTLELTLK**
	**132**	**149**	**1875.01**	**NSGTLELTLKDGVLTDAK**
	**132**	**151**	**2088.13**	**NSGTLELTLKDGVLTDAKGR**
	**142**	**149**	**818.43**	**DGVLTDAK**
	**142**	**151**	**1031.55**	**DGVLTDAKGR**
Ssa1p	35	47	1471.71	TTPSFVAFTDTER
	125	136	1327.66	MKETAESYLGAK
	137	153	1897.93	VNDAVVTVPAYFNDSQR
	158	169	1199.67	DAGTIAGLNVLR
	170	186	1787.99	IINEPTAAAIAYGLDKK
	218	233	1675.73	ATAGDTHLGGEDFDNR
	234	243	1274.69	LVNHFIQEFK
	249	255	833.41	DLSTNQR
	297	308	1526.74	ARFEELCADLFR
	326	339	1459.78	SQVDEIVLVGGSTR
	507	514	961.52	LSKEDIEK
Scw4p	**135**	**148**	**1533.69**	**GITYTPYESSGACK**
	149	167	2019.04	SASEVASDLAQLTDFPVIR
	**168**	**181**	**1686.78**	**LYGTDCNQVENVFK**

	189	207	2185.15	VFLGIYYVDQIQDGVNTIK
	292	308	1986.04	NTVAQDSGKWLLEQIQR
	301	308	1085.61	WLLEQIQR
	309	317	1064.52	VWTACDGKK
	370	378	909.43	ADGAYGVEK
	**370**	**386**	**1871.88**	**ADGAYGVEKYWGILSNE**
	379	386	981.47	YWGILSNE
Scw10p	**138**	**151**	**1576.66**	**GITYSPYNDDGSCK**
	152	170	2065.02	STAQVASDLEQLTGFDNIR
	**171**	**186**	**1822.90**	**LYGVDCSQVENVLQAK**
	321	343	2436.19	DVVITETGWPSKGDTYGEAVPSK
	**344**	**354**	**1131.60**	**ANQEAAISSIK**
	**355**	**372**	**2053.93**	**SSCGSSAYLFTAFNDLWK**
Tdh3p	47	53	835.37	YDSTHGR
	54	70	1882.93	YAGEVSHDDKHIIVDGK
	117	137	2207.11	VVITAPSSTAPMFVMGVNEEK
	117	143	2914.46	VVITAPSSTAPMFVMGVNEEKYTSDLK
	144	160	1705.86	IVSNASCTTNCLAPLAK
	161	184	2575.31	VINDAFGIEEGLMTTVHSLTATQK
	185	195	1297.63	TVDGPSHKDWR
	199	213	1374.72	TASGNIIPSSTGAAK
	232	246	1470.84	VPTVDVSVVDLTVK
	247	258	1481.78	LNKETTYDEIKK
	308	321	1752.79	LVSWYDNEYGYSTR
	322	332	1179.67	VVDLVEHVAKA
Tos1p	**355**	**367**	**1575.66**	**TLQYGDASCSCWK**
	**368**	**385**	**1943.87**	**TGCGEMDLFEILTAGSDK**
	**412**	**421**	**1051.54**	**AAVIFNSSDK**

Note:

a. Mass for singly charged peptide with Lys-D0 labeling.

b. Peptides were from trypsin digestion with up to three Lys or Arg miss-cleavage.

c. The underlined cysteines were carbamidomethyl modified with a 57.02 Da increase in mass.

d. Peptide sequences in bold were used for SILAC quantification.
