# Supplementary material for: Characterization of Cell Wall Proteins in Saccharomyces cerevisiae Clinical Isolates Elucidates Hsp150p in Virulence
Source: PLoS One. 2015 Aug 13;10(8):e0135174. doi: 10.1371/journal.pone.0135174 (PMC4535956; doi:10.1371/journal.pone.0135174)
Supplement: S2 Table — (PDF) [file pone.0135174.s003.pdf]

S2 Table: List of peptides from cytoplasmic proteins as the internal control for SILAC quantification.

Protein ID	sequence		$[M+H]^+$	Peptide sequence [b, c, d]
	start	end		
EF-1a	**6**	**20**	**1560.85**	**SHINVVVIGHVDSGK**
	31	37	805.40	CGGIDKR
	**38**	**44**	**894.49**	**TIEKFEK**
	**56**	**62**	**894.47**	**YAWVLDK**
	**56**	**64**	**1135.65**	**YAWVLDKLK**
	56	67	1491.83	YAWVLDKLKAER
	85	96	1355.71	YQVTVIDAPGHR
	85	100	1858.98	YQVTVIDAPGHRDFIK
	101	129	2923.45	NMITGTSQADCAILIIAGGVGEFEAGISK
	135	146	1326.75	EHALLAFTLGVR
	**147**	**154**	**884.56**	**QLIVAVNK**
	160	170	1436.72	WDESRFQEIVK
	165	170	763.44	FQEIVK
	**165**	**177**	**1582.85**	**FQEIVKETSNFIK**
	165	178	1710.94	FQEIVKETSNFIKK
	171	177	838.43	ETSNFIK
	185	210	2908.40	TVPFVPISGWNGDNMIEATTNAPWYK
	211	217	877.44	GWEKETK
	218	224	658.43	AGVVKGK
	223	245	2548.41	GKTLLEAIDAIEQPSRPTDKPLR
	225	245	2363.29	TLLEAIDAIEQPSRPTDKPLR
	**246**	**253**	**975.55**	**LPLQDVYK**
	254	264	1025.61	IGGIGTVPVGR
	**265**	**288**	**2430.33**	**VETGVIKPGMVVTFAPAGVTTEVK**
	**289**	**311**	**2550.20**	**SVEMHHEQLEQGVPGDNVGFNVK**
	320	328	976.46	RGNVCGDAK
	320	333	1527.73	RGNVCGDAKNDPPK
	**321**	**328**	**820.36**	**GNVCGDAK**
	**370**	**376**	**893.46**	**FDELLEK**

	370	379	1278.63	FDELLEKNDR
	370	380	1434.73	FDELLEKNDRR
	384	390	866.47	KLEDHPK
	**394**	**401**	**760.42**	**SGDAALVK**
	**402**	**421**	**2311.13**	**FVPSKPMCVEAFSEYPPLGR**
	**429**	**437**	**914.57**	**QTVAVGVIK**
Pdc1p	**37**	**44**	**996.48**	**IYEVEGMR**
	**128**	**151**	**2522.21**	**MSANISETTAMITDIATAPAEIDR**
	**180**	**209**	**3323.80**	**LLQTPIDMSLKPNDAESEKEVIDTILALVK**
	**213**	**224**	**1375.65**	**NPVILADACCSR**
	**210**	**224**	**1689.81**	**DAKNPVILADACCSR**
	**234**	**249**	**1777.96**	**LIDLTQFPAFVTPMGK**
	**233**	**249**	**1906.05**	**KLIDLTQFPAFVTPMGK**
	**259**	**273**	**1596.86**	**YGGVYVGTLSKPEVK**
	**305**	**315**	**1356.64**	**NIVEFHSDHMK**
	**333**	**342**	**1016.60**	**LLTTIADAAK**
	**343**	**352**	**1057.62**	**GYKPVAVPAR**
	**479**	**484**	**664.41**	**LIHGPK**
	**485**	**505**	**2388.20**	**AQYNEIQGWDHLSLLPTFGAK**
	**512**	**520**	**1006.48**	**VATTGEWDK**
	**512**	**525**	**1591.80**	**VATTGEWDKLTQDK**
	**535**	**554**	**2286.19**	**MIEIMLPVFDAPQNLVEQAK**
Pma1p	**100**	**109**	**1168.53**	**YGLNQMADEK**
	**100**	**115**	**1823.92**	**YGLNQMADEKESLVVK**
	**175**	**184**	**1057.64**	**TLANTAVVIR**
	**185**	**215**	**3259.67**	**DGQLVEIPANEVVPGDILQLEDGTVIPTDGR**
	**216**	**239**	**2652.31**	**IVTEDCFLQIDQSAITGESLAVDK**
	**240**	**252**	**1456.67**	**HYGDQTFSSSTVK**
	**240**	**253**	**1612.77**	**HYGDQTFSSSTVKR**
	**253**	**271**	**2013.98**	**RGEGFMVVTATGDNTFVGR**
	**254**	**271**	**1857.88**	**GEGFMVVTATGDNTFVGR**
	**272**	**278**	**686.42**	**AAALVNK**
	**429**	**435**	**863.50**	**SLKQYPK**
	**520**	**548**	**3254.47**	**GEGHWEILGVMPCMDPPRDDTAQTVSEAR**
	**538**	**548**	**1192.54**	**DDTAQTVSEAR**

**554**	**566**	**1302.75**	**VKMLTGDAVGIAK**
**910**	**918**	**1058.51**	**VSTQHEKET**

Note:

a. Mass for singly charged peptide with Lys-D0 labeling.

b. Peptides were from trypsin digestion with up to three Lys or Arg miss-cleavage.

c. The underlined cysteines were carbamidomethyl modified with a 57.02 Da increase in mass.

d. Peptide sequences in bold were used for SILAC quantification.
