# Supplementary material for: Characterization of Cell Wall Proteins in Saccharomyces cerevisiae Clinical Isolates Elucidates Hsp150p in Virulence
Source: PLoS One. 2015 Aug 13;10(8):e0135174. doi: 10.1371/journal.pone.0135174 (PMC4535956; doi:10.1371/journal.pone.0135174)
Supplement: S3 Table — (PDF) [file pone.0135174.s004.pdf]

S3 Table: The averaged relative quantitative levels of cell wall proteins analyzed by MassMatrix (MM) and MaxQuant (MQ).

Cell Wall	**YYC1**		**YYC2**		**YYC3**		**YYC38**	
Protein	MM	MQ	MM	MQ	MM	MQ	MM	MQ
**GPI-CWP**								
Ccw14p/Ssr1p	1.33±0.04	1.28±0.39	2.84±0.40	1.58±0.61	1.05±0.01	0.86±0.18	1.09±0.07	1.01±0.05
Crh1p	2.38±0.34*	2.17±0.61*	6.89±1.20*	4.80±2.44*	2.40±0.28*	1.88±0.25*	2.02±0.31*	2.16±0.52*
Crh2p/Utr2p	2.80#	3.09±1.40*	4.07±0.81	2.35±1.17	1.42±0.18	1.23±0.38	2.02±0.65	2.17±0.39*
Cwp1p	1.86±0.46*	1.65±0.65	2.17±0.55*	1.28±0.77	1.37±0.25	1.01±0.24	1.56±0.41	1.05±0.07
Ecm33p	2.38±0.30*	1.85±0.52*	2.94±0.64*	--	2.23±0.22*	--	1.42±0.34	1.25±0.25
Gas1p	1.52±0.30	1.36±0.39	2.41±0.45*	1.49±0.62	1.16±0.13	0.91±0.07	1.21±0.20	1.16±0.16
Gas3p	0.35±0.21*	0.43±0.23*	1.01±0.17	0.74±0.37	0.56±0.06	0.68±0.18*	0.66±0.15	0.70±0.19*
Gas5p	1.13±0.50	1.06±0.38	1.76±0.25	1.01±0.52	0.51±0.15*	0.76±0.13	0.71±0.30	0.68±0.22
Pst1p	10.19±1.85	8.02±0.59	15.88#	5.39±1.32	1.97±0.18	3.82±0.73	3.20#	3.71±0.18
**ASL-CWP**								
Cis3p/Pir4p	3.37±0.41*	3.40±0.82*	3.55±0.53*	2.17±1.10	2.71±0.63*	2.44±0.45*	2.15±0.62*	2.34±0.44*
Hsp150p/Pir2p	2.66±0.60*	3.21±1.39*	7.59±1.14*	4.90±2.24*	2.46±0.72*	2.22±0.26*	2.75±0.67*	3.05±0.56*
Pir1p	2.45±0.39*	2.80±0.82*	5.09±2.28*	2.42±1.12*	1.96±1.01	1.26±0.31	2.24±0.36*	2.21±0.51*
Scw4p	2.48±0.26*	2.13±0.65*	3.15±0.24*	1.83±0.81	0.90±0.84	1.39±0.18*	1.82±0.17*	1.81±0.29*
Scw10p	3.97±0.99	4.43±0.77*	10.60±2.26	6.56±2.82*	5.85±0.98	4.97±0.78*	5.39±2.06	4.53±0.85*
Tos1p	1.91±0.35	1.57±0.36*	3.06±0.37*	1.76±0.98	1.29±0.13	1.04±0.18	1.50±0.01	1.37±0.24*

*Asterisks indicate statistically significant differences from S288c.

No SD data available due to the single detection in 5 biological replicates.
